# Homocysteine and the risk of age-related macular degeneration: a systematic review and meta-analysis

**DOI:** 10.1038/srep10585

**Published:** 2015-07-21

**Authors:** Peirong Huang, Fenghua Wang, Birendra Kumar Sah, Junhai Jiang, Zhentian Ni, Jentso Wang, Xiaodong Sun

**Affiliations:** 1 Department of Ophthalmology, Shanghai First People’s Hospital, School of Medicine, Shanghai Jiao Tong University, Shanghai, China; 2 Eye Research Institute of Shanghai Jiao Tong University, Shanghai, China; 3 Department of Surgery, Ruijin Hospital, School of Medicine, Shanghai Jiao Tong University, Shanghai, China; 4 Division of Biostatistics, University of Texas Health Science Center, Houston, USA

## Abstract

Contrasting results have been reported regarding the associations between plasma total homocysteine (tHcy) and B vitamin levels and age-related macular degeneration (AMD) risk. Thus, we aimed to systematically evaluate these associations. Relevant case control studies in English were identified via a thorough search of the PubMed, Medline, and Embase databases from inception to June 2014. The results were pooled using Review Manager 5.2.1. Eleven studies (including 1072 cases and 1202 controls) were eligible for analysis of tHcy levels; additionally, 3 studies (including 152 cases and 98 controls) were eligible for analysis of folic acid and vitamin B_12_ levels. The cumulative results demonstrated that the plasma tHcy level among the AMD cases was 2.67 μmol/L (95% confidence interval [CI], 1.60-3.74) higher than that among the controls. In contrast, the vitamin B_12_ level among the AMD cases was 64.16 pg/mL (95% CI, 19.32-109.00) lower than that among the controls. Subgroup analyses showed that the folic acid level was 1.66 ng/mL (95% CI, 0.10-3.21) lower for the wet type. Together, the results demonstrated that AMD is associated with elevated tHcy levels and decreased vitamin B_12_ levels. Plasma tHcy may act as a modulator of the risk for AMD based on the current evidence.

Age-related macular degeneration (AMD) is the primary cause of permanent vision loss among individuals greater than 50 years of age in industrialised countries and affects 25-35 million people worldwide^1^,^2^,^3^. Generally, AMD is stratified into two stages, namely, early and late age-related maculopathy (ARM), according to the International Classification and Grading System designed by the International ARM Epidemiological Study Group^4^. Advanced (late) AMD can be further classified into non-neovascular (dry, atrophic) and neovascular (wet, exudative) types. Few therapeutic or preventative strategies are currently available for the dry type, which constitutes approximately 80% of all late AMD cases^5^. Anti-vascular endothelial growth factor (VEGF) therapy has demonstrated great benefit for the wet type, although issues, such as the need for repeated injections and non-responses, continue to occur^6^. Accordingly, new therapies are anticipated.

The exact pathogenesis of AMD remains poorly understood. Several risk factors have been suggested, including advanced age, Caucasian ethnicity, smoking, blue light irradiation, oxidative stress, and genetic factors. Recent epidemiological evidence has implicated a direct association between plasma total homocysteine (tHcy) levels and AMD risk. Plasma tHcy levels may be influenced by many factors. Nutritional factors, including serum vitamin B_6_, folic acid, and vitamin B_12_, are common and important regulators of plasma tHcy levels that can be modulated by diet, suggesting a simple homocysteine-lowering therapy. Hyperhomocysteinemia has been demonstrated to be an independent risk factor for cardiovascular disease (CVD) and atherosclerosis^7^. Interestingly, a link between AMD, atherosclerosis, and CVD has been observed^8^,^9^. Moreover, AMD patients exhibit an elevated cardiovascular risk profile and increased prospective CVD risk. These findings imply a common causal pathway among AMD, atherosclerosis and CVD and hyperhomocysteinemia may act as a common etiological role in these diseases, specifically in the induction of endothelial injury and atherosclerosis, both of which are involved in these diseases.

Many studies have been conducted to elucidate the association between AMD and homocysteine. However, the results were inconsistent^10^,^11^,^12^,^13^,^14^,^15^,^16^,^17^,^18^,^19^,^20^. Thus, we aimed to combine the current evidence to elucidate the relationship between serum tHcy, folic acid, and vitamin B_12_ levels and the risk of AMD.

## Results

### Study characteristics

**Figure 1:**
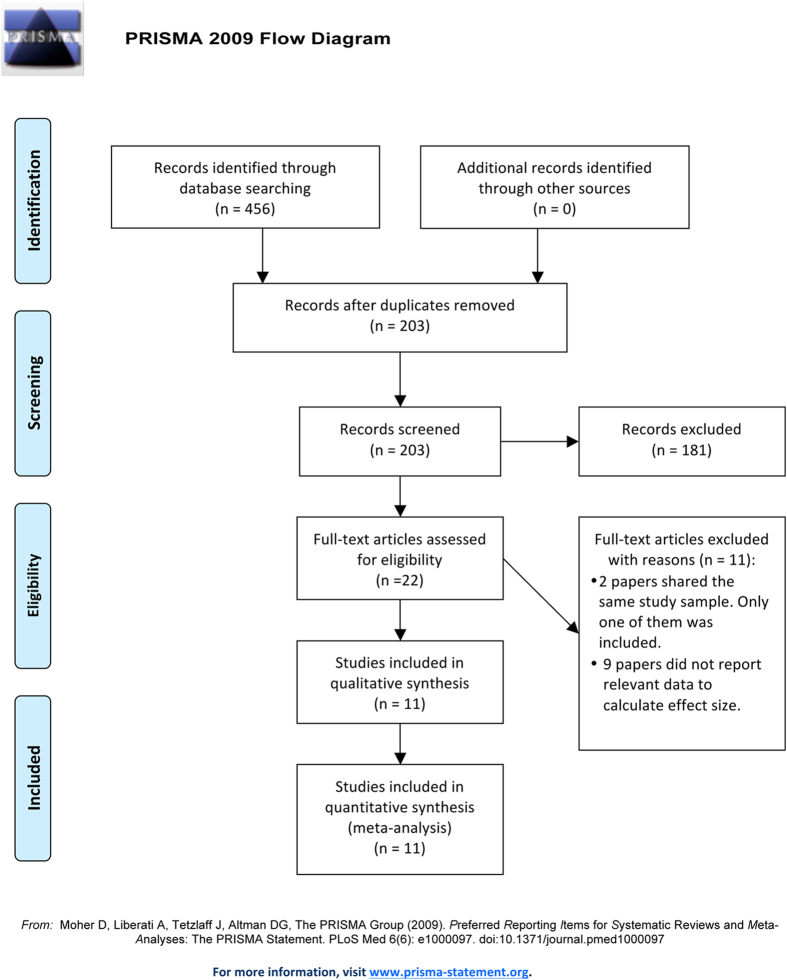
Flow chart of the study search and selection strategies.

A flow chart of the screening progress is shown in Fig. 1. Ultimately, 11 studies were included, among which 11 studies (including 1072 cases and 1202 controls) were eligible for analysis of tHcy levels, 3 studies (including 152 cases and 98 controls) were eligible for analysis of folic acid levels, and 3 (including 152 cases and 98 controls) were eligible for analysis of vitamin B_9_ levels. The number of studies that examined the association of vitamin B_6_ levels or a polymorphism of methylenetetrahydrofolate reductase (MTHFR), methionine synthase (MS), or cystathionine β-synthase (CBS) with AMD was less than two. Therefore, we did not perform a pooled analysis for these factors. Details regarding the included studies are presented in Table 1.

### Pooled analysis

#### Total homocysteine

**Figure 2:**
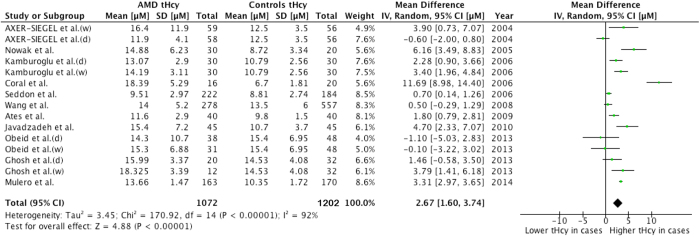
Forest plots for the association of the plasma tHcy levels with AMD.

The combined difference in the serum homocysteine levels of the eligible studies and the corresponding odds ratio (OR) and 95% confidence interval (CI) are shown in Fig. 2. The dots indicate the estimated mean differences (MDs), and the length of the lines indicates the associated 95% CI. The values to the right of the longitudinal line at 0 represent higher tHcy levels in the AMD patients, whereas the values to the left of the longitudinal line represent higher tHcy levels in the control subjects.

Overall, the pooled results showed that the serum tHcy level among the AMD patients was 2.67 μmol/L higher than that among the controls; this difference was significant (96% CI, 1.60-3.74) with extreme heterogeneity (*I*^*2*^=92%, P<0.00001). Sensitivity analyses indicated that this result was not excessively influenced by any particular study.

#### Folic acid

**Figure 3:**
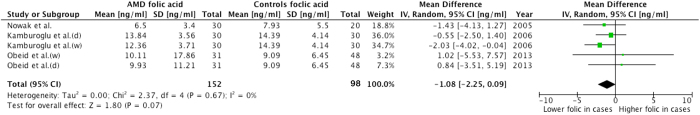
Forest plots for the association of the serum folic acid levels with AMD.

Figure 3 presents the forest plot of the serum folic acid levels in the AMD cases and the controls. This figure can be interpreted in the same manner as Fig. 2 except that the results are expressed in ng/mL. The values to the left of the longitudinal line at 0 represent lower serum folic acid levels in the AMD patients, whereas the values to the right of the longitudinal line indicate lower serum folic acid levels in the controls.

The combined results revealed no difference in the serum folic acid levels between the AMD patients and the controls. The mean difference was −1.08 ng/mL (95% CI, −2.25-0.09), and no between-study heterogeneity was observed (*I*^*2*^=0%, P=0.67).

#### Vitamin B_12_

**Figure 4:**
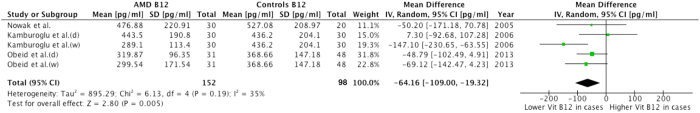
Forest plots for the association of the serum vitamin B_12_ levels with AMD.

The differences in the pooled plasma vitamin B_12_ levels between the AMD cases and the controls (Fig. 4) can also be interpreted as described above, with the exception that the results are expressed in pg/mL. The values to the left of the vertical line at 0 represent lower serum vitamin B_12_ levels in the AMD patients, whereas the values to the right of the longitudinal line indicate lower serum vitamin B_12_ levels in the controls.

The pooled results showed that the mean serum vitamin B_12_ level among the AMD patients was 64.16 pg/mL (95% CI, 19.32-109.00) lower than that among the controls, and moderate heterogeneity was observed (*I*^*2*^ = 35%, P = 0.19).

### Subgroup analyses

Factors influencing the plasma tHcy levels or the AMD status may bias the pooled results. Therefore, we conducted subgroup analyses according to the AMD stage (late AMD or any AMD), clinical subtype of late AMD (wet AMD or dry AMD), AMD diagnostic method (ophthalmic or photographic), and age and gender differences between the cases and controls.

Only one study (Wang *et al.* 2008) combined both early and late AMD (any AMD) and revealed a small and non-significant association with the tHcy level (0.50 μmol/L, 95%CI, −0.29-1.29). Studies including only late AMD cases revealed a high and significant association with the estimated tHcy level (2.87 μmol/L, 95%CI, 1.74-4.00). Studies of the folic acid and vitamin B_12_ levels included only late AMD cases. Therefore, subgroup analysis was not performed in this respect.

The wet and dry AMD types differ significantly with respect to the natural disease course. Regarding tHcy levels, subgroup analyses showed a higher level of homocysteine among the wet AMD patients than among all AMD patients (pooled mean difference 4.14 μmol/L, 95% CI, 2.78-5.51 vs. 0.75 μmol/L, 95% CI, −0.95-2.45). However, the dry type was associated with an insignificant elevation of the plasma tHcy level, in contrast to the result for all AMD patients. Regarding folic acid, the analysis of only the exudative AMD patients revealed significantly lower plasma folic acid levels in these patients but not in all patients (1.66 ng/mL, 95% CI, 0.10-3.21 vs. 1.08 ng/mL, 95% CI, −0.09-2.25). Regarding vitamin B_12_ levels, subgroup analyses revealed an insignificant difference in levels between the dry AMD patients and the controls, in contrast to the results for all AMD patients. In summary, only exudative AMD was associated with significantly higher tHcy levels and lower folic acid and vitamin B_12_ levels.

Three studies (Coral *et al.* 2006; Seddon *et al.* 2006; Wang *et al.* 2006) based on photographic grading yielded a slightly higher association between the tHcy level and any AMD type (3.74, 95% CI, 0.69-6.80). The studies based on ophthalmic examination revealed a lower association between tHcy levels and late AMD (2.47 μmol/L, 95% CI, 1.49-3.44). All studies on folic acid and vitamin B_12_ levels utilised ophthalmic examination. Thus, no subgroup analysis was performed on these factors.

The level of plasma homocysteine was approximately 10% higher in healthy males than in healthy females. Two studies revealed a gender difference between the cases and controls. One of these two studies (Coral *et al.* 2006), which included a higher percentage of males among the cases, demonstrated higher tHcy levels in the AMD patients, whereas the other study (Obeid *et al.*), which included a lower percentage of males among the cases, demonstrated a non-significant difference in the tHcy levels between the two groups (11.69 μmol/L, 95% CI, 8.98-14.40, −0.49 μmol/L, 95% CI, −2.93-1.96, respectively).

The incidence of AMD increases with age. In all the eligible studies, the cases and controls were either age-matched or were not significantly different with respect to gender. Furthermore, the mean age of the participants in all of the included studies was greater than 60 years. Therefore, subgroup analysis was not performed for age.

The results of the analysis of publication bias, which was evaluated using the fail-safe number (N_fs_), are shown in Table 2. Additional caution should be taken regarding the subtype analyses of the association of folic acid with wet/dry AMD and of vitamin B_12_ with dry AMD, as the corresponding N_fs_ values were smaller than the number of included studies, suggesting marked publication bias.

## Discussion

We primarily examined the association of the tHcy level with the risk of AMD in this meta-analysis. Briefly, our study suggested that the plasma tHcy level was elevated but the vitamin B_12_ level was reduced in AMD patients compared with healthy controls. This association was found to be enhanced for the wet AMD type but insignificant for the dry AMD type.

The overall meta-analyses of the eligible studies confirmed a significantly elevated plasma level of tHcy in the AMD patients. This elevation was significant for the late AMD patients but not for the study including both early and late AMD patients. This trend suggested that the plasma tHcy level may increase with disease progression. Thus, plasma tHcy level may serve as a biomarker of AMD with which to monitor disease status. Moreover, the abnormal metabolism of tHcy may play an etiological role in the development of AMD, particularly for the exudative type. These findings suggest a future direction of research.

The mechanisms underlying hyperhomocysteinemia in AMD remain unclear, but several reasons are implied based on increasing evidence. First, oxidative stress may play a major role. The retina is particularly susceptible to reactive oxygen species (ROS) because of 1) direct exposure to light, 2) high consumption of oxygen, and 3) high concentrations of polyunsaturated fatty acids in the photoreceptors^21^. Homocysteine is an active oxidising agent that can exacerbate oxidative stress-induced injury^22^. Second, increased serum homocysteine levels can cause direct epithelial damage and retinal pigment epithelium (RPE) junction disruption^23^, both of which can lead to neovascularisation. Third, elevated homocysteine levels can promote inflammatory processes that ultimately induce atherosclerosis^24^. The mechanisms noted above all contribute to the underlying pathogenesis of AMD and atherosclerosis.

Sensitivity analyses of the tHcy levels were performed by excluding one study at a time to demonstrate the effect of each study on the overall pooled results. The results remained within the CI, which indicated that the results were stable. The N_fs_, reflecting publication bias, was quite large, indicating that the publication bias was minor.

The classifications of AMD were not uniform in the included studies. One study used the Wisconsin grading system^25^, two studies used the AREDS classification^26^, and the other studies simply used the terms “Dry AMD” and “Wet AMD” without clarification (Table 1). However, the definitions of neovascular AMD are similar between each classification system. Thus, the use of different classification systems would not affect these results. Alternatively, some patients in the “intermittent AMD group” based on the AREDS classification who exhibited geographic atrophy may have been included in the “late AMD group” according to other classification systems. This discrepancy may weaken the associations between elevated homocysteine levels and AMD risk. Moreover, the classification of the control groups may also contain discrepancies. As defined in the AREDS classification system, patients displaying drusen with a maximum size < 63 μm and a total diameter < 125 μm are included in the “No AMD group”; however, some of these patients would be included in the “early stage AMG group” according to other classification systems. This discrepancy would also weaken these associations. In this regard, among the currently available systems, the AREDS classification system is highly recommended in future studies because of its good repeatability and accuracy.

The current pooled data showed a non-significant difference in the folic acid levels between the cases and controls, although subgroup analyses showed a significant difference between the AMD wet group and controls. Regarding vitamin B_12_, the AMD patients exhibited significantly lower levels. This association was stronger in the wet AMD subgroup but was not detected in the dry AMD subgroup. The difference in the results between the wet and dry AMD cases and the controls may be attributed to their apparently distinct disease characteristics.

The causal role of folic acid and vitamin B_12_ in AMD cannot be well established based on our combined data due to the small number of studies included. Thus, conclusions based on these analyses require further supportive results. Despite the small number of previously published articles, our findings were in accordance with two high-quality studies^27^,^28^. The Blue Mountains Eye Study, which reported the 10-year incidence of AMD, revealed that an elevated serum level of total homocysteine increased the probability of developing AMD by approximately 30% but that an increased serum concentration of vitamin B_12_ decreased the probability of developing AMD by approximately 30%^29^. The other high-quality randomised placebo-controlled trial with 7.3 years of follow-up, the Women’s Antioxidant and Folic Acid Cardiovascular Study (WAFACS), reported that the incidence of AMD doubled among individuals with folate or vitamin B_12_ deficiency at baseline and that daily supplementation with vitamins B_6_/B_9_/B_12_ reduced the probability of developing AMD by 35-40%^30^. This beneficial effect emerged in the second year of treatment.

The concept that vitamin B supplementation prevents the development of AMD is of great interest; however, this treatment is far from being recommended for clinical use at this stage^31^. The role of other nutritional supplements has been studied extensively, and the results have been promising. The original Age-Related Eye Disease Study (AREDS) showed that daily supplementation with vitamin C, vitamin E, β-carotene, and zinc reduced the risk of progression by 25% in 5 years^32^. The AREDS-2 treatment paradigm substituted carotene for lutein and zeaxanthin and also showed benefits^33^. Whether B vitamins may serve as auxiliary dietary supplements requires further investigation. In addition, we should seriously consider the findings from studies of cardiovascular disease. Although observational studies showed elevated levels of homocysteine and decreased levels of B vitamins, homocysteine-lowering interventions failed to reduce the risk of CVD in randomised placebo-controlled trials^34^. In conclusion, the limited studies on B vitamins may cause bias in the pooled results regarding folic acid and vitamin B_12_; thus, these results should be interpreted cautiously. It is also too early to recommend any treatment for clinical use to prevent AMD development.

There are some limitations of our study that should be considered during its interpretation. First, the small number of included studies on B vitamins may lead to publication bias and selective reporting. Second, AMD is a multi-factorial disease that is both genetically and environmentally influenced. An ideal study design would adjust for covariates. Our studies were not adjusted due to the limitations of the original study design. Important covariates, including age, gender, ethnicity, smoking status, atherosclerotic cardiovascular disease, and glucose levels, were adjusted in a portion of the included studies. Third, the distinct classification systems of AMD subtypes may weaken the difference in the homocysteine and B vitamin levels between late AMD or dry AMD patients and controls, although the “wet AMD type” was nearly consistently defined. The AREDS classification system is recommended for future studies. In short, further prospective multi-centre RCTs using a clarified united classification of AMD, larger samples, and various ethnicity that adjust for confounding risk factors may overcome the aforementioned limitations of previous data collection and analysis methods.

Despite the shortcomings mentioned above, the current study revealed preliminarily useful clinical results by providing evidence supporting a potential intervention for both types of AMD. Supplementation of vitamin B or folic acid for AMD prevention/control is biologically plausible based on the currently available evidence. Additional randomised clinical trials in different races are anticipated to aid in determining the efficiency and safety of homocysteine-lowering therapy.

## Methods

Our meta-analysis strictly complies with the Preferred Reporting Items for Systematic Reviews and Meta-analyses (PRISMA) statement^35^.

### Literature search

Two investigators (P.R. Huang and J.H. Jiang) were involved in the literature search of PubMed, Medline, and Embase from inception to June 2014. The search terms used were “age-related macular degeneration”, “homocysteine”, “pyridocine”, “folic acid”, “cobalamin”, “vitamin B_6_”, “vitamin B_9_”, “vitamin B_12_”, “methylenetetrahydrofolate reductase (MTHFR)”, “methionine synthase (MS)”, and “cystathionine β-synthase (CBS)” in various combinations. Related citations in PubMed, along with the references of each retrieved study, were also examined. The searches were restricted to studies published in the English language and performed in humans. Common agreement between two researchers was a prerequisite for the final inclusion of a qualified article. If two or more studies were based on the same cohort, the more definitive study was included.

### Inclusion and exclusion criteria

Articles were only included under the following conditions: (1) case control study consisting of a laboratory assessment of (2) plasma total homocysteine, (3) vitamin B (including vitamin B_6_, B_9_, and B_12_), or (4) a polymorphism of MTHFR, MS, or CBS. Studies were excluded if they were non-controlled, in the non-English literature, or reported as abstracts from academic conferences.

### Study selection

From the initial 456 relevant articles identified in the databases, 22 full texts were assessed for eligibility. Two reports shared the same study sample, so only one study was included. Nine studies did not report relevant data to enable calculation of the effect size. Ultimately, eleven studies were included.

### Data extraction

Two authors (X.D. Sun and P.R. Huang) independently selected the qualified studies according to the inclusion and exclusion criteria listed above. The following data were collected: (1) the homocysteine levels; (2) the vitamin B levels; (3) MTHFR, MS, and CBS polymorphisms; and (4) characteristics of the included studies, such as the first author, the year and geographical location of the study, mean age, gender ratio, type of AMD, AMD grading method, and AMD classification and grading system. Other relevant data that were missing from the reports were acquired from the respective authors.

### Statistical analysis

The data were collected and analysed using RevMan software (version 5.2.1, The Nordic Cochrane Centre, Copenhagen, Denmark). The MD and 95% CI were calculated separately for tHcy, folic acid, and cobalamin. The difference between the AMD patients and the controls was displayed using a forest plot. The *I*^*2*^ statistic (ranging from 0 to 100%) was applied to quantify between-study heterogeneity not attributed to chance (*I*^*2*^ = 0-25%, no heterogeneity; *I*^*2*^ = 25-50%, moderate heterogeneity; *I*^*2*^ = 50-75%, large heterogeneity; and *I*^*2*^ = 75-100%, extreme heterogeneity). A random-effects model was employed in this study.

Publication bias was assessed using the fail-safe number (N_fs_), and the statistical threshold was 0.05. A calculated N_fs_ smaller than the number of included studies in a given comparison was considered to indicate significant publication bias. We calculated the significance of N_fs_ using the formula N_fs_0.05 = (∑Z/1.64)^2^−k, where k represents the number of included studies^36^,^37^,^38^,^39^.

## Additional Information

**How to cite this article**: Huang, P. *et al*. Homocysteine and risk of age-related macular degeneration: a systematic review and meta-analysis. *Sci. Rep.* **5**, 10585; doi: 10.1038/srep10585 (2015).

## Figures and Tables

**Table 1:** Study design and baseline characteristics of the included studies.

**Study**	**Year and location**	**Study design**	**Study content**	**AMD type**	**Number of patients**	**Age, years mean (SD)**	**PCT of males (%)**	**AMD grading method**	**AMD classification and grading system**
Axer-Siegel *et al.*^10^	2004, Israel	Clinic-based, prospective, cross-sectional	tHcy	group 1. wAMD	group 1. n = 59	78 ± 8.4	42.37	Ophthalmologist examination	NA
				group 2. dAMD	group 2. n = 58	76.3 ± 8.4	41.38		
				group 3. control	group 3. n = 56	76.3 ± 8.4	48.21		
Nowak *et al.*^11^	2005, Poland	Clinic-based, case-control	tHcy	group 1. wAMD	group 1. n = 30	66.2 ± 3.6	NA	Ophthalmologist examination	NA
			B_9_, B_12_	group 2. control	group 2. n = 20	65.8 ± 5.2	NA		
Coral *et al.*^12^	2006, India	Clinic-based, case-control	tHcy	group 1. wAMD	group 1. n = 16	66 (51–82)	68.75	Photographic grading	AREDS classification system
			GSH, tSH	group 2. control	group 2. n = 20	62 (55–75)	40		
Kamburoglu *et al.*^13^	2006, Turkey	Clinic-based, prospective, cross-sectional	tHcy	group 1. wAMD	group 1. n = 30	69.7 ± 7.2	50	Ophthalmologist examination	NA
			B_9_, B_12_	group 2. dAMD	group 2. n = 30	69.9 ± 6.8	43.33		
				group 3. control	group 3. n = 30	69.9 ± 7.0	36.67		
Seddon *et al.*^14^	2006, USA	Population-based, cross-sectional, case-control	tHcy	group 1. Late AMD	group 1. n = 222	71 ± 5.1	45	Photographic grading	AREDS classification system
				group 2. control	group 2. n = 184	67 ± 4.2	36		
Wang *et al.*^15^	2008, Australia	Population-based, case-control	tHcy	group 1. Early and late AMD	group 1. n = 278	75.6 ± 8.5	NA	Photographic grading	Wisconsin age-related maculopathy grading system
				group 2. control	group 2. n = 557	74.9 ± 7.9	NA		
Ates *et al.*^16^	2009, Turkey	Clinic-based, cross-sectional	tHcy	group 1. wAMD	group 1. n = 40	63.3 ± 5	45	Ophthalmologist examination	NA
				group 2. control	group 2. n = 40	61 ± 4	NA		
Javadzadeh *et al.*^17^	2010, Iran	Clinic-based, case-control	tHcy	group 1. wAMD	group 1. n = 45	71 ± 7	40	Ophthalmologist examination	NA
				group 2. control	group 2. n = 45	69 ± 5	40		
Ghosh *et al.*^18^	2013, India	Clinic-based, case-control	tHcy	group 1. wAMD	group 1. n = 12	67.4 ± 6.5	41.67	Ophthalmologist examination	NA
				group 2. dAMD	group 2. n = 20		45		
				group 3. control	group 3. n = 32	66.5 ± 5.9	43.75		
Obeid *et al.*^19^	2013, Germany	Clinic-based, case-control	tHcy	group 1. wAMD	group 1. n = 31	78 (67–86)	51.61	Ophthalmologist examination	NA
			B_9_, B_12_	group 2. dAMD	group 2. n = 38	77 (68–86)	26.32		
				group 3. control	group 3. n = 48	74 (60–81)	48.94		
Mulero *et al.*^20^	2014, Spain	Clinic-based, cross-sectional, case-control	tHcy	group 1. wAMD	group 1. n = 163	71 ± 7.3	49	Ophthalmologist examination	NA
				group 2. control	group 2. n = 170	71 ± 6.7	52	Ophthalmologist examination	NA

AMD: age-related macular degeneration; PCT: percentage; tHcy: total homocysteine; NA: not available; AREDS: Age-Related Eye Disease Study; GSH: glutathione; tSH: thiol content.

**Table 2:** Fail-safe number.

**Comparison**	**N_fs_**
tHcy with any AMD	1292.03
tHcy with late AMD	1239.02
tHcy with wet AMD	900.91
tHcy with dry AMD	9.47
Folic acid with late AMD	3.81
Folic acid with wet AMD	1.17
Folic acid with dry AMD	-1.68
Vitamin B_12_ with late AMD	20.97
Vitamin B_12_ with wet AMD	10.88
Vitamin B_12_ with dry AMD	−0.63

N_fs_: fail-safe number; tHcy: total homocysteine; AMD: age-related macular degeneration.
